# Comparison of Smartphone Ownership, Social Media Use, and Willingness to Use Digital Interventions Between Generation Z and Millennials in the Treatment of Substance Use: Cross-Sectional Questionnaire Study

**DOI:** 10.2196/13050

**Published:** 2019-04-17

**Authors:** Brenda L Curtis, Robert D Ashford, Katherine I Magnuson, Stacy R Ryan-Pettes

**Affiliations:** 1 Psychology-Addictions Treatment Research Center University of Pennsylvania Philadelphia, PA United States; 2 Substance Use Disorders Institute University of the Sciences Pennsylvania, PA United States; 3 Department of Psychology and Neuroscience Baylor University Waco, TX United States

**Keywords:** social media, mHealth, substance use treatment, digital health, recovery, social networking sites, substance use disorder

## Abstract

**Background:**

Problematic substance use in adolescence and emerging adulthood is a significant public health concern in the United States due to high recurrence of use rates and unmet treatment needs coupled with increased use. Consequently, there is a need for both improved service utilization and availability of recovery supports. Given the ubiquitous use of the internet and social media via smartphones, a viable option is to design digital treatments and recovery support services to include internet and social media platforms.

**Objective:**

Although digital treatments delivered through social media and the internet are a possibility, it is unclear how interventions using these tools should be tailored for groups with problematic substance use. There is limited research comparing consumer trends of use of social media platforms, use of platform features, and vulnerability of exposure to drug cues online. The goal of this study was to compare digital platforms used among adolescents (Generation Zs, age 13-17) and emerging adults (Millennials, age 18-35) attending outpatient substance use treatment and to examine receptiveness toward these platforms in order to support substance use treatment and recovery.

**Methods:**

Generation Zs and Millennials enrolled in outpatient substance use treatment (n=164) completed a survey examining social media use, digital intervention acceptability, frequency of substance exposure, and substance use experiences. Generation Zs (n=53) completed the survey in July 2018. Millennials (n=111) completed the survey in May 2016.

**Results:**

Generation Zs had an average age of 15.66 (SD 1.18) years and primarily identified as male (50.9%). Millennials had an average age of 27.66 (SD 5.12) years and also primarily identified as male (75.7%). Most participants owned a social media account (Millennials: 82.0%, Generation Zs: 94.3%) and used it daily (Millennials: 67.6%, Generation Zs: 79.2%); however, Generation Zs were more likely to use Instagram and Snapchat, whereas Millennials were more likely to use Facebook. Further, Generation Zs were more likely to use the features within social media platforms (eg, instant messaging: Millennials: 55.0%, Generation Zs: 79.2%; watching videos: Millennials: 56.8%, Generation Zs: 81.1%). Many participants observed drug cues on social media (Millennials: 67.5%, Generation Zs: 71.7%). However, fewer observed recovery information on social media (Millennials: 30.6%, Generation Zs: 34.0%). Participants felt that social media (Millennials: 55.0%, Generation Zs: 49.1%), a mobile phone app (Millennials: 36.9%, Generation Zs: 45.3%), texting (Millennials: 28.8%, Generation Zs: 45.3%), or a website (Millennials: 39.6%, Generation Zs: 32.1%) would be useful in delivering recovery support.

**Conclusions:**

Given the high rates of exposure to drug cues on social media, disseminating recovery support within a social media platform may be the ideal just-in-time intervention needed to decrease the rates of recurrent drug use. However, our results suggest that cross-platform solutions capable of transcending generational preferences are necessary and one-size-fits-all digital interventions should be avoided.

## Introduction

Substance use and substance use disorder (SUD) among adolescents and young adults is a major public health concern in the United States. The high rates of substance use coupled with significant unmet treatment needs and alarming rates of recurrence of use are concerning. Recent estimates suggest that 2.0 million adolescents aged 12-17 years and 8.3 million young adults aged 18-25 years used illicit substances in 2017. Among these users, 1.0 million of the adolescent users and 5.2 million of the young adult users were identified as needing SUD treatment. However, only 184,000 of the former and 641,000 of the latter received the required treatment [1]. Strikingly, among those who complete treatment, research suggests that 60%-70% will have a recurrence of use within 90 days after a treatment episode and 85%, within 1 year following treatment [2-4].

These rates of substance use, the unmet need for treatment, and the recurrence of use following treatment suggest that there is a critical need for effective ways to increase service utilization and to make recovery tools available to adolescents and young adults. One way to increase the use of treatment services for SUD among this population is to leverage technologies that are very engaging and already widely used. Given the widespread use of the internet via smartphones and the ubiquitous use of social media, a viable option is to design digital treatments and recovery supports to include internet and social media platforms.

According to new research by the Pew Institute, almost all adolescents and young adults in the United States have access to a smartphone (95% of those aged 13-17 years, 94% of those aged 18-29 years, and 89% of those aged 30-49 years) [5,6]; research suggests that these smartphone owners use the internet extensively. For example, 89% of adolescents aged 13-17 years report using the internet via a mobile device almost constantly or several times a day. Among adults, 89% access the internet daily and 31% access the internet constantly [7]. Noteworthily, visiting social media platforms appears to be important to adolescents and young adults using the internet. Recent results by the Pew Institute [5,8] found that over 92% of adolescents and 88% of adults in the United States use social media platforms. Teens frequently use social media platforms, with 70% using them more than once daily, 38% using them multiple times an hour, and 16% using them near constantly [9]. Among adolescents, the most frequently used social media platforms are YouTube (85%), Instagram (72%), and Snapchat (69%) [5]. In contrast, YouTube and Facebook are used most often among adults (those aged 18-29 years: 91% for YouTube and 81% for Facebook; those aged 30-39 years: 85% for YouTube and 78% for Facebook) [8]. Further, the majority of adult social media users visit these sites very often, with 74% reporting use of Facebook several times a day or at least once a day and 46% reporting use of YouTube several times a day or at least once a day [8].

Designing digital treatments to include the internet and social media platforms is not a novel idea. In fact, in populations of youth with diabetes and obesity, treatment modalities and recovery support through social media were shown to be feasible and effective [10-12]. More specifically, in a sample of 20 adolescents between the ages of 12 and 16 years with type 1 diabetes, those who used a mobile health app with a social networking component showed an improvement in blood glucose monitoring [10]. In addition, preliminary findings in a sample of 13 youth with obesity indicated that social media groups are an acceptable and effective adjunct to obesity treatment [11]. Social media as an intervention has also been used to target sexual health and tobacco cessation. For example, a clustered randomized control trial with 1578 young adults found Facebook an effective medium to disseminate health education [13]. In addition, Facebook was an effective intervention medium for smoking cessation in a sample of 79 young adults [14]. More specifically, this sample of young adults receiving a smoking cessation intervention via Facebook reduced its cigarette consumption by 50% [14].

Although this research demonstrates that the use of the internet and social media to deliver interventions is not new, there are two gaps in the relevant literature. One, our review failed to find reports of formative work that informed the selection of social media platforms and features based on consumer trends. The features included in digital interventions and the selected platforms are likely to vary by population and consumer trends. Popularity of digital platforms and usage trends appear to ebb and flow. For example, until recently, Facebook was the most visited social media site among youth and young adults [5]. Additionally, factors that are related to technology acceptance and social media use are influenced by age [15,16]. Two, there is a dearth of knowledge about the online behavior and preferences for digital treatment and recovery programs in populations enrolled in substance use treatment programs. Although findings of research on adults who use illicit substances are available [17], our review of the literature revealed a lack of knowledge about the use of digital platforms among adolescents attending outpatient substance use treatment. Substance use researchers have begun to explore the use of mobile phones to support recovery from SUDs among adolescents [18]. As researchers move beyond the use of the basic capabilities of mobile phones (eg, texting) and take advantage of more dynamic features of digital and social media platforms [19], it is important to first understand the online behavior and preferences for this type of treatment and recovery support among adolescents. Further, since the development of treatment programs for adolescents tend to trickle down from adult research, it is important to have knowledge of access to mobile phones and the use of digital and social media platforms among adolescents in substance use treatment as compared to other generations in substance use treatment. A better understanding of preferences and use of these platforms across generations of substance users may help with the efforts to tailor technology and digital media platform-based recovery programs. The technology-acceptance model for social media use [15] and evidence showing age as a moderator of mobile health service adoption [16] suggest evident differences in platform use and preferences for key features across generations because younger populations are better able to adapt to novel operating procedures, which increase the perceived ease of use.

Comparing Generation Z and Millennials may be particularly informative. Millennials were coming of age during the proliferation of technology into homes and during the first major introduction of social media into popular culture [20]. Thus, Millennials have adopted technology and social media more than the older generations [21]. On the other hand, Generation Zs were born into a social media culture, positively impacting key variables related to digital and social media acceptance, including perceived ease of use, subjective norm, trust, and reduced risk [15,16]. The purpose of this study is to fill these gaps in the literature by examining and comparing characteristics of various digital platforms used among adolescents (Generation Zs) and young adults (Millennials) attending outpatient substance use treatment and to examine the degree to which these generations are receptive to using these platforms to support substance use treatment and recovery.

## Methods

### Participants

Adolescents (Generation Zs) and young adults (Millennials) enrolled in outpatient substance use treatment programs in the Southwest and Northeast and regions of the United States, respectively, participated in this study. The requirements to participate in the survey were enrollment in an outpatient program at the time of the survey (all participants), age of 18-35 years (Millennials) or 13-17 years (Generation Zs), no intellectual or developmental disability (all participants), and willingness to provide informed consent or assent to participate. All study procedures were approved by the University of Pennsylvania Human Subjects Review Board and the UT Health San Antonio and Baylor University Institutional Review Boards.

Data for the current study comprise two collated datasets (Generation Zs and Millennials) using an identical survey instrument. The Millennial dataset (n=111) is a subset (those aged between 18 and 35 years only) of a larger sample (n=259) previously reported [17]. The Generation Z dataset has not been previously reported. Following recruitment and data collection, cleaned data were combined into a single data set for analysis.

### Procedure

Recruitment of Generation Zs was completed in July 2018, while data from the Millennials were gathered in May 2016. All participants attending outpatient treatment for an SUD completed self-administered, in-person, paper-and-pencil surveys. All participants were invited to participate in this study before, after, or between scheduled group treatment sessions. Participation in this study was voluntary, and it was made clear to all participants that agreeing to be part of this study would not have an impact on their treatment or on any legal proceeding that may have required them to be in treatment. Participants provided individual consent to participate, and where applicable (due to age), participants’ parents or legal guardians also provided consent for their child to participate. The survey took 10-15 minutes to complete, and no identifiable information was recorded in order to protect participant privacy.

### Measures

The survey used is identical to the survey described by Ashford et al [17] and included questions about technology ownership and use (eg, mobile phone, internet, and social media) and soliciting acceptability and willingness to participate in SUD interventions delivered via digital platforms (Multimedia Appendix 1).

#### Technology Ownership and Social Media Use

To facilitate comparison across studies in the substance use literature, technology ownership and use were measured using modified questions developed by McClure and colleagues [22] and widely used by the Pew Research Center, a leading authority on trends in mobile phone technology and internet and social media use. We measured social media account ownership and use via novel questions following a structure similar to that developed previously by McClure [22]. All ownership and use questions relied upon self-reported information from participants.

#### Acceptability and Willingness to Participate in a Digital Intervention

We measured participant willingness to use online platforms for interventions that promote positive recovery outcomes through responses to the following locally developed items: (1) “Do you think social media would be a good place to receive information to help you avoid relapse?” (binary; yes/no), (2) “Would you join an online support group to help you during your recovery?” (binary; yes/no), (3) “Would you join a Facebook support group to help you during your recovery?” (binary; yes/no), (4) “Would you sign up to receive text messages to help you during your recovery?” (binary; yes/no), and (5) “Would you use an app placed on your phone to help your recovery from alcohol or substance use?” (binary; yes/no). We also asked participants to identify the platform they would most like to use in order to access a digital support program to aid during recovery (website, social media, texting, and digital app), and if they would allow their social media accounts to be monitored to help prevent relapse (binary; yes/no).

#### Frequency of Exposure to Drug Cues and Recovery Information

Participant exposure frequency to drug cues (eg, text, still imagery, or video content related to illicit or licit substances) and recovery cues (eg, text, still imagery, or video content related to recovery and wellness) on social media was measured via responses to the following locally developed items: (1) “How often have you seen drug cues—things that made you want to use drugs on social media?” (Likert scale: 1 [always] to 5 [never]), (2) “How often have you seen recovery information on social media?” (Likert scale: 1 [always] to 5 [never]), and (3) “Have you posted information on social media about being in recovery?” (binary; yes/no).

#### Substance Use Preferences and Experiences

Participants’ preferences and experiences related to past substance use were collected through a combination of the Alcohol Use Disorder Identification Test - Alcohol Consumption Questions (AUDIT-C) [23], the Drug Abuse Screen Test (DAST-10) [24], and a single self-report question asking participants if a substance was their preferred substance of use (alcohol, opioids, cocaine, amphetamines, marijuana, or other). For this study, we did not use standardized scoring of the AUDIT-C or the DAST-10. Participants were recruited from SUD treatment settings and were presumably already provided an SUD diagnosis. As we were primarily interested in participant substance use preferences and experiences, rather than the diagnosis, we reported individual question responses for each generation and compared response rates for each.

### Data Analysis

Data from Millennial participants are a subset of a larger sample reported previously (n=259) [17], although data from the AUDIT-C and DAST-10 were not previously reported. Generation Z responses were entered into a data-monitoring system using double entry. One research assistant entered the data while checking for mismatches and out-of-range values. A different research assistant then entered the same data again. The two entries were compared via a computer that identified mismatches. When mismatches were identified, the person who entered the data checked the original survey to determine the correct value.

All analyses were performed using SPSS V24.0 (IBM Corp, Armonk, NY). Descriptive statistics were calculated for the total sample as well as the Generation Z and Millennial subsamples. Pearson chi-square tests were used to compare technology ownership, use, potential use, and willingness over the two generational groups. No data were excluded from participants meeting the inclusion criteria of this study.

## Results

### Participants

Demographic information for all participants is shown in Table 1. Overall, participants (n=164) had a mean age of 23.78 (SD 7.06) years, primarily identified as male (67.7%), and were unemployed or current students (75.6%). Most participants self-reported cannabis as a primary substance of use (78.7%), followed by alcohol (34.1%), opioids (23.8%), cocaine (13.4%), and amphetamines (6.7%).

Generation Zs (aged 12-17 years; n=53) had a mean age of 15.66 (SD 1.18) years, primarily identified as male (50.9%), and were unemployed or current students (88.7%). Most Generation Zs self-reported cannabis as a primary substance of use (98.1%), followed by alcohol (32.1%), opioids (11.3%), cocaine (9.4%), and amphetamines (7.5%).

Millennials (aged 18-35 years; n=111) had a mean age of 27.66 (SD 5.12) years, primarily identified as male (75.7%), and were unemployed or current students (69.4%). Most Millennials self-reported cannabis as a primary substance of use (69.4%), followed by alcohol (35.1%), opioids (29.7%), cocaine (15.3%), and amphetamines (6.3%).

### Technology Ownership and Use

The majority of both generations owned a mobile phone (Millennial [hereafter referred to as M]: 93.7%, Generation Z [hereafter referred to as Z]: 90.6%) that was identified as a smartphone (M: 71.2%, Z: 90.6%), used it regularly (M: 96.4%, Z: 98.1%), and had either a pay-as-you-go (M: 64.9%, Z: 28.3%) or yearly contract (M: 27.0%, Z: 52.8%). Chi-square testing (Table 2) showed that smartphone ownership and contract type were related to generation, with Generation Zs more likely to own a smartphone than Millennials and Millennials more likely to use pay-as-you-go contracts than Generation Zs. Most participants from both generations had changed their number at least once (M: 70.2%, Z: 75.4%), while a small percentage had changed their number four or more times (M: 11.7%, Z: 15.1%).

The majority of participants from both generations used the internet regularly (M: 86.5%, Z: 96.2%), with most accessing the internet via a mobile phone (M: 72.1%, Z: 50.9%). Chi-square tests found that the way in which the internet was accessed was related to generation, with Millennials more likely to use a mobile phone than Generation Zs, and Generation Zs more likely to use a computer at home or another method of access.

The majority of all participants also regularly used a computer (M: 56.8%, Z: 54.7%), email (M: 72.1%, Z: 54.7%), and text messaging (M: 93.7%, Z: 94.3%). The generation was found to be related to only the regular use of email, but the Millennials were more likely to use email than Generation Zs.

### Social Media Ownership and Use

Most participants owned a social media account (M: 82.0%, Z: 94.3%) and used it daily (M: 67.6%, Z: 79.2%) or weekly (M:15.3%, Z: 15.1%). Prominent social media platforms used included Facebook (M: 80.2%, Z: 66.0%), Instagram (M: 61.3%, Z: 83.0%), Twitter (M: 27.0%, Z: 26.4%), Google+ (M: 29.7%, Z: 22.6%), and Snapchat (M: 27.0%, Z: 79.2%). Chi-square tests found that use of Facebook, Instagram, and Snapchat was related to participant generation, with Millennials more likely to use Facebook and Generation Zs more likely to use Instagram and Snapchat.

**Table 1:** Participant demographic characteristics.

Characteristic		Combined sample (N=164), n (%)	Millennial sample (n=111), n (%)	Generation Z sample (n=53), n (%)
Age (years), mean (SD)		23.78 (7.06)	27.66 (5.12)	15.66 (1.18)
**Gender**				
	Female	53 (32.3)	27 (24.3)	26 (49.1)
	Male	111 (67.7)	84 (75.7)	27 (50.9)
**Race**				
	Black	82 (50.0)	70 (63.1)	12 (22.6)
	Nonblack	82 (50.0)	41 (36.9)	41 (77.4)
Ethnicity (Latino)		65 (39.6)	24 (21.6)	41 (77.4)
**Education level**				
	Did not complete high school	87 (53.0)	34 (30.6)	53 (100.0)
	High school graduate or GED^a^	64 (39.0)	64 (57.7)	0 (0.0)
	Two-year degree or more	13 (8.0)	13 (11.7)	0 (0.0)
**Employment status**				
	Employed	40 (24.4)	34 (30.6)	6 (11.3)
	Unemployed/studying	124 (75.6)	77 (69.4)	47 (88.7)
**Primary substance of use**				
	Alcohol	56 (34.1)	39 (35.1)	17 (32.1)
	Opiates	39 (23.8)	33 (29.7)	6 (11.3)
	Cocaine	22 (13.4)	17 (15.3)	5 (9.4)
	Amphetamine	11 (6.7)	7 (6.3)	4 (7.5)
	Cannabis	129 (78.7)	77 (69.4)	52 (98.1)

^a^GED: general educational development.

Social media was used in a variety of ways by all participants who reported using social media (Table 2). Common activities that social media was used for included instant messaging (M: 55.0%, Z: 79.2%), seeing updates about others (M: 53.2%, Z: 69.8%), watching videos from others (M: 56.8%, Z: 81.1%), reading the news and other information (M: 45.0%, Z: 62.3%), staying in touch with friends and family (M: 64.0%, Z: 86.8%), and finding entertaining content (M: 51.4%, Z: 98.1%). Each of the activities mentioned above was found to be related to the generation, with Generation Zs more likely to use social media to instant message, see updates about others, watch videos from others, find news and information, stay in touch with friends and family, and find entertaining content.

### Drug Cues and Recovery Support on Digital Platforms

Most participants had seen drug cues on social media (M: 67.5%, Z: 71.7%), with 22.5% of Millennials seeing drug cues either always or very often and 34.0% of Generation Zs seeing them at the same frequency. Conversely, a higher percentage of both generations had never seen recovery information on social media (M: 30.6%, Z: 34.0%), with 16.2% of Millennials and 26.4% of Generation Zs seeing recovery information either always or very often. Less than one-third of both generations reported having previously posted recovery information on their social media (M: 26.1%, Z: 26.4%). Chi-square tests found that the generation was related to seeing recovery information on social media platforms, with Generation Zs more likely to report seeing recovery information always or very often and Millennials more likely to report seeing recovery information sometimes or rarely.

Although a majority of Millennials believe current social media platforms could be used to prevent recurrence of use (50.5%), a little more than one-third of Generation Zs felt the same way (37.7%), but this value was not statistically significant. When asked what type of platform should be used to deliver recovery support, participants felt that social media (M: 55.0%, Z: 49.1%), a mobile phone app (M: 36.9%, Z: 45.3%), text messages (M: 28.8%, Z: 45.3%), or a website (M: 39.6%, Z: 32.1%) would be useful. Participants’ beliefs in the usefulness of texting as a platform were found to be related to generation, with Generation Zs more likely to believe a texting platform would be useful. Less than half the participants from either generation reported a willingness to consent to social media monitoring to support their recovery (M: 36.9%, Z: 24.5%).

**Table 2:** Technology ownership and usage characteristics by generation.

Ownership and usage characteristics			Millennial sample (n=111), n (%)	Generation Z sample (n=53), n (%)	Chi-square test	
					X^2^ (*df*)	*P* value
**Mobile phone ownership**					0.5 (1)	.47
	Yes		104 (93.7)	48 (90.6)		
	No		7 (6.3)	5 (9.4)		
**Smartphone ownership**					9.5 (1)	.002^a^
	Yes		79 (71.2)	48 (90.6)		
	No		32 (28.8)	5 (9.4)		
**Mobile phone regular use**					0.4 (1)	.55
	Yes		107 (96.4)	52 (98.1)		
	No		4 (3.6)	1 (1.9)		
**Mobile phone contract type**					19.4 (2)	<.001^b^
	Pay as you go		72 (64.9)	15 (28.3)		
	Yearly contract		30 (27.0)	28 (52.8)		
**Changed phone number**					2.1 (4)	.72
	Never		31 (27.9)	13 (24.5)		
	One time		33 (27.9)	19 (35.8)		
	Two times		16 (14.4)	8 (15.1)		
	Three times		18 (16.2)	5 (9.4)		
	Four or more times		13 (11.7)	8 (15.1)		
**Internet access**					13.4 (3)	.01^a^
	Via mobile phone		80 (72.1)	27 (50.9)		
	Via computer at home		12 (10.8)	18 (34.0)		
	Via other method		4 (3.6)	6 (11.3)		
**Internet regular use**					3.7 (1)	.056
	Yes		96 (86.5)	51 (96.2)		
	No		15 (13.5)	2 (3.8)		
**Computer regular use**					0.1 (1)	.81
	Yes		63 (56.8)	29 (54.7)		
	No		48 (43.2)	24 (45.3)		
**Email regular use**					4.8 (1)	.03^a^
	Yes		80 (72.1)	29 (54.7)		
	No		31 (27.9)	24 (45.3)		
**Text message regular use**					0.0 (1)	.87
	Yes		104 (93.7)	50 (94.3)		
	No		7 (6.3)	3 (5.7)		
**Social media account ownership**					4.5 (1)	.03^a^
	Yes		91 (82.0)	50 (94.3)		
	No		20 (18.0)	3 (5.7)		
**Social media use frequency**					4.3 (3)	.23
	Daily		75 (67.6)	42 (79.2)		
	Weekly		17 (15.3)	8 (15.1)		
	Monthly		1 (0.9)	0 (0.0)		
	Do not use regularly		18 (16.2)	3 (5.7)		
**Social media used for…**						
	**Share photos or videos**				0.0 (1)	.95
		Yes	77 (69.4)	37 (69.8)		
		No	34 (30.6)	16 (30.2)		
	**Instant message**				9.1 (1)	.003^a^
		Yes	61 (55.0)	42 (79.2)		
		No	50 (45.0)	11 (20.8)		
	**Share updates about self**				2.3 (1)	.13
		Yes	56 (50.5)	20 (37.7)		
		No	55 (49.5)	33 (62.3)		
	**Meet new people**				2.1 (1)	.15
		Yes	51 (45.9)	18 (34.0)		
		No	60 (54.1)	35 (66.0)		
	**See updates about others**				4.1 (1)	.04^a^
		Yes	59 (53.2)	37 (69.8)		
		No	52 (46.8)	16 (30.2)		
	**Watch videos others post**				9.3 (1)	.002^a^
		Yes	63 (56.8)	43 (81.1)		
		No	48 (43.2)	10 (18.9)		
	**News and information**				4.3 (1)	.04^a^
		Yes	50 (45.0)	33 (62.3)		
		No	61 (55.0)	20 (37.7)		
	**Stay in touch with friends and family**				9.1 (1)	.002^a^
		Yes	71 (64.0)	46 (86.8)		
		No	40 (36.0)	7 (13.2)		
	**Find funny or entertaining content**				35.2 (1)	<.001^b^
		Yes	57 (51.4)	52 (98.1)		
		No	54 (48.6)	1 (1.9)		
**Social media platforms used**						
	**Facebook**				3.9 (1)	.049^a^
		Yes	89 (80.2)	35 (66.0)		
		No	22 (19.8)	18 (34.0)		
	**Twitter**				0.0 (1)	.93
		Yes	30 (27.0)	14 (26.4)		
		No	81 (73.0)	39 (73.6)		
	**Google+**				0.9 (1)	.34
		Yes	33 (29.7)	12 (22.6)		
		No	78 (70.3)	41 (77.4)		
	**Instagram**				7.5 (1)	.005^a^
		Yes	68 (61.3)	44 (83.0)		
		No	43 (38.7)	9 (17.0)		
	**Tumblr**				0.4 (1)	.51
		Yes	7 (6.3)	2 (3.8)		
		No	104 (93.7)	51 (96.2)		
	**Pinterest**				0.1 (1)	.71
		Yes	8 (7.2)	3 (5.7)		
		No	103 (92.8)	50 (94.3)		
	**Snapchat**				39.7 (1)	<.001^b^
		Yes	30 (27.0)	42 (79.2)		
		No	81 (73.0)	11 (20.8)		
	**LinkedIn**				3.0 (1)	.09
		Yes	6 (5.4)	0 (0.0)		
		No	105 (94.6)	53 (100.0)		
	**Myspace**				4.0 (1)	.045
		Yes	8 (7.2)	0 (0.0)		
		No	103 (92.8)	53 (100.0)		
**Seen drug cues on social media**					4.5 (2)	.49
	Always/very often		25 (22.5)	18 (34.0)		
	Sometimes/rarely		50 (45.0)	20 (37.7)		
	Never		26 (23.4)	11 (20.8)		
**Seen recovery information on social media**					18.5 (2)	.002^a^
	Always/very often		18 (16.2)	14 (26.4)		
	Sometimes/rarely		49 (44.1)	13 (24.5)		
	Never		34 (30.6)	18 (34.0)		
**Post recovery information**					0.0 (1)	.97
	Yes		29 (26.1)	14 (26.4)		
	No		82 (73.9)	39 (73.6)		
**Social media should be used to prevent relapse**					2.3 (1)	.13
	Yes		56 (50.5)	20 (37.7)		
	No		55 (49.5)	33 (62.3)		
**Type of platform to deliver relapse-prevention support**						
	**Website**				0.9 (1)	.35
		Yes	44 (39.6)	17 (32.1)		
		No	67 (60.4)	36 (67.9)		
	**Social media**				0.5 (1)	.48
		Yes	61 (55.0)	26 (49.1)		
		No	50 (45.0)	27 (50.9)		
	**Texting**				4.3 (1)	.04^a^
		Yes	32 (28.8)	24 (45.3)		
		No	79 (71.2)	29 (54.7)		
	**Mobile phone app**				1.04 (1)	.31
		Yes	41 (36.9)	24 (45.3)		
		No	70 (63.1)	29 (54.7)		
**Consent to social media monitoring to support recovery**					2.5 (1)	.11
	Yes		41 (36.9)	13 (24.5)		
	No		70 (63.1)	40 (75.5)		

^a^Significant at *P*=.05.

^b^Significant at *P*<.001.

### Substance Use Preferences and Experiences

Most participants reported alcohol intake frequency of monthly or less (M: 67.5%, Z: 81.1%) as well as never having heavy drinking episodes (M: 52.3%, Z: 60.4%). Most participants from either generation had engaged in illicit substance use at some point in their life (M: 55.9%, Z: 64.2%), with less than half reporting polysubstance use (M: 31.5%, Z: 47.2%).

Most participants felt they could stop using substances at any time they wanted (M: 53.2%, Z: 77.4%), and this was related to generation, with Generation Zs being more likely than Millennials to believe they could stop using at any time.

Participants reported a variety of experiences related to their substance use (Table 3). More prevalent experiences for both generations included their family complaining about their substance use (M: 55.9%, Z: 81.1%), guilty feelings (M: 48.6%, Z: 45.3%), engagement in illegal activities (M: 48.6%, Z: 32.1%), or experiencing withdrawal symptoms (M: 41.4%, Z: 32.1%). Family complaining about substance use and engagement in illegal activities were related to generation, with Generation Zs more likely to have experienced complaining family members and Millennials more likely to have engaged in illegal activities.

**Table 3:** Alcohol and other substance use trends and experiences.

Trends and experiences				Millennial sample (n=111), n (%)		Generation Z (n=53), n (%)		Chi-square test	
								X^2^ (*df*)	*P* value
**Alcohol intake frequency**								8.3 (4)	.14
	Never		38 (34.2)		22 (41.5)				
	Monthly or less		37 (33.3)		21 (39.6)				
	2-4 times a month		21 (18.9)		9 (17.0)				
	2-3 times a week		10 (9.0)		0 (0.0)				
	4 or more times a week		5 (4.5)		1 (1.9)				
**Heavy drinking frequency**								1.9 (4)	.76
	Never		58 (52.3)		32 (60.4)				
	Less than monthly		35 (31.5)		16 (30.2)				
	Monthly		11 (9.9)		3 (5.7)				
	Weekly		5 (4.5)		1 (1.9)				
	Daily or almost daily		2 (1.8)		1 (1.9)				
**Illicit substance use**								1.0 (1)	.31
	Yes		62 (55.9)		34 (64.2)				
	No		49 (44.1)		19 (35.8)				
**Polysubstance use**								3.8 (1)	.05
	Yes		35 (31.5)		25 (47.2)				
	No		76 (68.5)		28 (52.8)				
**Can stop using substances at any time^b^**								9.9 (1)	.007^a^
	Yes		59 (53.2)		41 (77.4)				
	No		46 (41.4)		12 (22.6)				
**Experiences related to substance use**									
	**Blackouts/flashbacks**						0.9 (1)		.34
		Yes		33 (29.7)		12 (22.6)			
		No		78 (70.3)		41 (77.4)			
	**Guilty feeling^b^**						1.8 (1)		.41
		Yes		54 (48.6)		24 (45.3)			
		No		54 (48.6)		29 (54.7)			
	**Family complaints**						10.0 (1)		.002^a^
		Yes		62 (55.9)		43 (81.1)			
		No		49 (44.1)		10 (18.9)			
	**Neglected family members**						1.3 (1)		.25
		Yes		44 (39.6)		26 (49.1)			
		No		67 (60.4)		27 (50.9)			
	**Engagement in illegal activity**						4.0 (1)		.045^a^
		Yes		54 (48.6)		17 (32.1)			
		No		57 (51.4)		36 (67.9)			
	**Withdrawal symptoms**						1.3 (1)		.25
		Yes		46 (41.4)		17 (32.1)			
		No		65 (58.6)		36 (67.9)			
	**Medical problems**						0.5 (1)		.46
		Yes		22 (19.8)		8 (15.1)			
		No		89 (80.2)		45 (84.9)			

^a^Significant at *P*<.05.

^b^Sample size varies slightly due to missing data.

## Discussion

### Overview

These data provide an examination of digital platform use and willingness to receive treatment and recovery information via social media among a sample of Generation Zs attending outpatient treatment for an SUD compared to Millennials attending outpatient substance use treatment. Further, these data provide an examination of exposure to drug cues juxtaposing recovery information online and on social media. The results of this study demonstrate an important first step in examining user trends, experiences, and preferences to develop digital interventions that are tailored to demographic trends in use of digital mediums and preferred digital features among a substance-using population. Results of this study in conjunction with the follow-up formative work examining responses to key survey items have the potential to offer prescriptive programming for developers of digital recovery tools.

### Principal Results

Unsurprisingly, most Generation Zs and Millennials owned a smartphone, which is consistent with the national rates [5]. Compared to Millennials, Generation Zs were more likely to own a smartphone that was part of a “contract” plan. One explanation for this difference is that individuals under the age of 18 years are more likely to have a mobile phone that is covered as part of a single-family contract [25]. Additionally, results showed that Generation Zs were more likely to have dedicated internet access and Millennials mainly relied on their mobile phone to access the internet. An explanation for this difference is that it is more likely that individuals under the age of 18 years are attending schools that have computers with internet access and are more likely to be living at home with a parent or guardian who is able to provide access to multiple forms of technology [26]. Further, results showed that Millennials were more likely to use email than Generation Zs. One explanation for this difference is that email communication was the primary method for rapid communication as Millennials were coming of age [27], whereas texting and social media features were the primary method for rapid communication among Generation Zs [28].

Social media use dominates both age groups, with most respondents having a social media account that was used daily. However, the preferred social media platform differed between Generation Zs and Millennials, with Generation Zs being more likely to use Instagram and Snapchat and Millennials being more likely to use Facebook. These findings are consistent with current national trends [5] and extend previous findings by demonstrating that generational differences in preferred social media platforms can be generalized to adolescents and young adults in treatment for an SUD. Interestingly, while both generations used social media at high rates, Generation Zs used certain features of social media platforms more than Millennials (eg, instant messaging, seeing updates about others, watching videos from others, finding news and information, and finding entertaining content). This finding suggests that Generation Zs may be more likely to use such features if they were included in a digital intervention.

### Implications for Designing Digital Interventions

These findings provide important information for developers interested in designing digital treatment and recovery programs for adolescents and young adults with problematic patterns of substance use. Findings that a large percentage of Generation Zs and Millennials in substance use treatment use social media suggests that the use of social media platforms to deliver treatment and recovery-related information would allow practitioners to meet these populations where they are. The most common digital platforms used for disseminating treatment and recovery information are mobile phone apps [19,29]. Results of this study suggest that general dissemination of information via apps may not reach the intended audience. Millennials are not likely to find mobile apps helpful. 

When asked what platform would be most helpful to receive recovery support, Generation Zs and Millennials rated social media above other platforms: 49% of Generation Zs and 55% of Millennials thought it would be helpful to receive relapse-prevention support via social media. This response rate is encouraging, considering that the remaining participants likely include a mix of at least four types of people: those who do not need relapse prevention by any method of delivery; those not interested in relapse prevention, in general; those who truly would not find recovery support through social media helpful; and those with privacy concerns. In support of our belief that participants may have privacy concerns related to recovery support delivered on social media, only 25.5% of Generation Zs and 36.9% of Millennials reported they would consent to social media monitoring to support their recovery. Our survey did not provide participants an opportunity to describe reasons for dissenting on either of these questions. Follow-up focus group data are needed to better understand Generation Zs’ and Millennials’ perceptions, acceptability, risk, and benefit. It is possible that an interactive dialogue could result in more participants agreeing on the helpfulness of recovery information delivered on social media and to allow their account to be monitored.

Results also showed there is unfavorable variance in the rates of exposure to drug cues and recovery-related information among both generations. The gap between exposure to drug cues and recovery-related information presents a unique opportunity to use social media platforms as an ideal location for digital interventions—both primary (eg, dissemination of treatment and recovery-related information) and tertiary (eg, reducing drug-related information) interventions. In total, 70%-75% of adolescents and young adults are on social media platforms multiple times a day, and 16% reported being on these platforms “near constantly” throughout the day [9]. For individuals with an SUD, exposure to drug cues online could serve as a trigger for continued or recurrent use. Immediate access to recovery supports online and within a social media platform may be the ideal just-in-time intervention necessary to decrease rates of recurrence of use.

Importantly, the high use of technology and digital platforms, along with the differences in the type and preference of specific platforms and activities, underscores the importance of program developers in conducting formative research to determine the preferred “location” at which the targeted population would like to receive interventions. For example, if the target audience is adolescents, the results of this study suggest that leveraging existing platforms such as Snapchat or Instagram may be ideal. However, if the target audience is young adults, leveraging Facebook may be more ideal. Further, the extant literature suggests that if the target audience cuts across Generation Zs and Millennials, leveraging YouTube may be ideal [5].

Although we were unable to find prior formative work on the characteristics and pattern of digital media use among adolescents in substance use treatment, results of this study and prior work with adults in treatment for substance use suggest that the use of a digital platform varies by age group [17,22]. Thus, we further recommend that program developers design tools and interventions that are multi- and cross-platform as a default and carefully consider using individual platforms only when there is a specific targeted audience in mind.

Development of digital interventions should be informed by trends in “user features.” Differential use of user features will allow for an informed design and dissemination of intervention programs that are more likely to be adopted by the target audience. For example, if the target audience is adolescents, the data suggest that a texting platform solution would be preferred. Similarly, results suggest that using instant messaging functions may be more impactful for targeting adolescents, while it may be less beneficial for targeting Millennials. Further, Generation Z may respond to recovery messages formatted in the way of news as well as exaggerated messages with cartoons that use humor to teach important skills. Additionally, developers should consider delivering therapeutic content and skills that have been produced by youth by using camera phones and that are capable of evoking relatable and realistic presentation of images. Apart from sharing photos or videos and staying in touch with friends and family, Millennials did not overwhelmingly report the use of any one social media feature. This suggest that although Millennials are online and most use social media like Facebook, they may be most interested in social media-based recovery tools that are less dynamic and include more static content like sharing videos and photos and posting messages with updates. This interpretation is consistent with research showing that older generations are more likely to adopt mobile health services that do not include a lot of operating procedures [16].

Results offer other useful information related to intervention content for digital-based intervention developers. For example, while Generation Zs reported higher rates of family complaints about their substance use as a negative experience, Millennials reported higher rates of criminal activity as negative experiences related to their substance use. Interventions should seek to incorporate this information, perhaps, including more family-oriented content for Generation Zs while incorporating decisional balance activities that help adults explore the pros and cons of substance use for Millennials. Generation Zs were also more likely to believe that they could stop using substances at any time. This may be explained by a shorter length of substance use overall, with fewer (if any) unsuccessful cessation attempts. Digital interventions designed for this generation may also benefit from motivational interviewing techniques [30], as those with an SUD are not more likely to have the ability to quit at will as a factor of age [31].

### Limitations

Several limitations of this study should be noted, as they may impact the interpretation and generalizability of the findings. There was limited overlap in data-collection periods between the two generations (Generation Z in 2018 and Millennials in 2016), and it is possible that there were changes in the popularity of certain platforms or feature use in this time frame. However, it is important to note that our results are consistent with current national trends [5-8] and provide a statistical test of observed generational differences. Additionally, the different locations where the data were collected (Generation Z data collected in the Southwestern United States and Millennial data collected in the Northeastern United States) may have captured regional (geographic) differences in addition to or instead of a difference between the generational cohorts. However, while research has suggested that geographic differences in social media use and technology ownership exist between rural, urban, and suburban areas, they have significantly declined in the last decade [21]. Finally, an overwhelming majority of participants reported using marijuana. Given the national trends in legalization of marijuana, a study with the power to conduct analyses by the type of illicit drug may provide additional information. It is possible that report of online exposure to drug information and openness to social media-based interventions are related to social attitudes about particular substances.

### Future Directions

In addition to the recommendations for researchers developing digital interventions as outlined above, these data point to the next steps in generating key formative research needed to develop dynamic digital interventions capable of delivering just-in-time treatment and recovery supports. With emerging data showing that social media language is related to diagnosis [32] and emerging machine learning techniques capable of predicting items such as county-level binge drinking rates [33], the design of a digital intervention will soon capitalize on the ability to deliver just-in-time interventions by monitoring language used by users of social media. In this study, a low percentage of Generation Zs and Millennials agreed to have their social media accounts monitored for delivering a treatment program or preventing a recurrence of use. Additional research is needed in this area, as research on older adults with problematic substance use has shown that monitoring and tracking digital behavior and activity are acceptable if respondents could control the features of the device or the service monitoring their actions [34,35]. It is likely that with follow-up questions on this topic, Generation Zs and Millennials would reveal similar results and, perhaps, guidance for how to include such a feature in a digital intervention. Future research should also identify the specific factors influencing the decision to allow monitoring, as it is possible that other reasons exist that can be mitigated through purposeful design and enhanced privacy features.

### Conclusions

To our knowledge, this study is the first study to compare two generational groups of clients attending treatment for an SUD in terms of the characteristics of technology, internet, and social media use as well as social media exposure to both drug cues and recovery information. Although mobile phone ownership (including smartphones) and the use of internet and social media platforms are high among both generations, the frequency of use and preference for user functions were different. Developers and practitioners may use findings from technology ownership and research to better inform digital interventions to improve adoption and efficacy and reduce attrition. Although the contrasts between generational cohorts suggest that specific features may be more impactful for a certain generation, such as texting platforms for Generation Zs, the overall future interventions would be well positioned to create cross-platform solutions that can be used across digital platforms, including texting, social media, websites, and mobile phone apps. One-size-fits-all digital interventions should be avoided, as cross-platform solutions are likely able to transcend generational preferences and increase adoption. Importantly, data from this study provide support for the need for digital interventions. The significant differences in the exposure to drug cues and recovery-related information on social media present as an immediate opportunity for intervention developers to immediately improve SUD treatment and recovery outcomes for both Generation Zs and Millennials.

## Appendix

Multimedia Appendix 1 Social media usage survey.

## Abbreviations

AUDIT-C: Alcohol Use Disorder Identification Test - Alcohol Consumption Questions
DAST-10: Drug Abuse Screen Test
SUD: substance use disorder
