# Supplementary material for: Comparison of Smartphone Ownership, Social Media Use, and Willingness to Use Digital Interventions Between Generation Z and Millennials in the Treatment of Substance Use: Cross-Sectional Questionnaire Study
Source: J Med Internet Res. 2019 Apr 17;21(4):e13050. doi: 10.2196/13050 (PMC6492066; doi:10.2196/13050)
Supplement: Multimedia Appendix 1 [file jmir_v21i4e13050_app1.pdf]

Thank you for participating in this University of Pennsylvania anonymous survey. We would like to examine the Internet and cell phone use of people attending outpatient alcohol and substance use treatment programs.

First, some basic questions about you....

1) How old are you? __________

2) Do you identify as:
☐ Female ☐ Male ☐ Transgender ☐ Intersex ☐ Other

3) Are you of Latino origin or descent, such as Mexican, Puerto Rican, Cuban?
☐ Yes ☐ No

4) Please specify your ethnicity: (Check all that apply)
☐ White
☐ Black or African American
☐ Native American or American Indian
☐ Asian / Pacific Islander
☐ Other

5) What is your marital status?
☐ Single, never married
☐ Married or domestic partnership
☐ Widowed
☐ Divorced
☐ Separated

6) What is the highest degree or level of school you have completed?
☐ Did not complete high school
☐ High school graduate or GED certificate
☐ Two year associate degree
☐ Four year college or university degree (Bachelors)
☐ Postgraduate or professional degree, including master's, doctorate, medical or law degree (e.g., MA, MS, PhD, MD, JD)

7) Are you currently:
☐ Employed
☐ Out of work and looking for work
☐ Out of work but not currently looking for work
☐ Unable to work
☐ A homemaker
☐ A student
☐ Retired

8) Last year, what was your total family income from all sources, before taxes?
☐ Less than $10,000 ☐ $10,000 to $29,999 ☐ $30,000 to $49,999 ☐ Over $50,000

<u>Now, some questions about your Cellphone and Internet Use</u>

9) Do you regularly (weekly) use a mobile/cell phone?
   ☐ Yes ☐ Yes, but not weekly ☐ No

10) Do you own this phone?
   ☐ Yes ☐ No

   a) Is this phone a Smartphone?
      ☐ Yes ☐ No

   b) What type of contract do you have for your cell phone?
      ☐ Pay-as-you-go ☐ Yearly contract ☐ I do not have a mobile/cell phone

   c) How often have you changed mobile/cell phones in the past year?
      ☐ Never ☐ One time ☐ 2 times ☐ 3 times ☐ More than 3 times

   d) Do you have unlimited calls on your mobile/cell phone plan?
      ☐ Yes ☐ No ☐ I do not have a mobile/cell phone

   e) Do you have unlimited text on your mobile/cell phone plan?
      ☐ Yes ☐ No ☐ I do not have a mobile/cell phone

11) Do you regularly (weekly) **send and receive text messages**?
   ☐ Yes ☐ Yes, but not weekly ☐ No

12) Do you regularly (weekly) use **email**?
   ☐ Yes ☐ Yes, but not weekly ☐ No

13) Do you regularly (weekly) use the **Internet (go online)**?
   ☐ Yes ☐ Yes, but not weekly ☐ No

14) **How** do you typically access the Internet (go online)?
   ☐ I use my cellphone or smartphone
   ☐ I go online where I live
   ☐ I go online at the library I go online at a friend or family members home
   ☐ I go online at church or a community center
   ☐ Other
   ☐ I don’t go online

15) Do you regularly (weekly) use a **computer**?
   ☐ Yes ☐ Yes, but not weekly ☐ No

16) Do you have a **social media account**? (ex: Facebook, Twitter)
   ☐ Yes ☐ No

17) How **often** do you use **social media** (ex: Facebook, Twitter)

☐	☐	☐	☐	☐
Daily	Weekly	Monthly	Yearly	I don't use social media

18) What **do** you do on **social media**? (Check all that apply)
- ☐ Share photos or videos
- ☐ Instant message
- ☐ Share updates about yourself
- ☐ Meet new people
- ☐ See updates about others
- ☐ Watch videos others post
- ☐ News and information
- ☐ Stay in touch with friends and family
- ☐ Find funny or entertaining content
- ☐ I don't use social media

19) Which **social media accounts do you have**? (Check all that apply)
- ☐ Facebook
- ☐ Twitter
- ☐ Google+ (Google Plus)
- ☐ Instagram
- ☐ Tumblr
- ☐ Pinterest
- ☐ Snapchat
- ☐ LinkedIn
- ☐ MySpace
- ☐ YikYak
- ☐ Other: ____
- ☐ I don't use social media

20) How often have you **seen drug cues—**things that made you want to use drugs **on social media**?

☐	☐	☐	☐	☐	☐
Always	Very often	Sometimes	Rarely	Never	I don't use social media

21) How often have you seen **recovery information** on **social media**?

☐	☐	☐	☐	☐	☐
Always	Very often	Sometimes	Rarely	Never	I don't use social media

22) Have you **posted** information on social media about being in **recovery**?

☐ Yes ☐ No

23) Do you think social media would be a good place to **receive** information to help you avoid relapse?

☐ Yes ☐ No

We are in the process of developing an online program to help people while they are in outpatient treatment. We would like to know the best way to provide this program to <u>someone like you</u>.

24) How should we provide this online program? (check all you would use)
☐ Website ☐ Social Media ☐ Texting ☐ Cell phone app

a) Would you join an online support group to help you during your recovery?
☐ Yes ☐ No

b) Would you join a Facebook support group to help you during your recovery?
☐ Yes ☐ No

c) Would you sign up to receive text messages to help you during your recovery?
☐ Yes ☐ No

d) Would you use an app placed on your phone to help your recovery from alcohol or substance use?
☐ Yes ☐ No

25) Would you allow your social media account(s) to be monitored if it could prevent you from relapsing?
☐ Yes ☐ No

<u>Finally, please tell us about your past alcohol and drug use</u>

26) What are your drugs of choice?
☐ Alcohol
☐ Opiates
☐ Cocaine
☐ Amphetamines
☐ Marijuana
☐ Other:______________________________

27) How often do you have a drink containing alcohol?
☐ Never
☐ Monthly or less
☐ 2-4 times a month
☐ 2-3 times a week
☐ 4 or more times a week

28) How many standard drinks containing alcohol do you have on a typical day?
☐ None
☐ 1 or 2
☐ 3 or 4
☐ 5 or 6
☐ 7 to 9
☐ 10 or more

29) How often do you have six or more drinks on one occasion?

☐ Never
☐ Less than monthly
☐ Monthly
☐ Weekly
☐ Daily or almost daily

30) How long have you been in your current outpatient treatment program?____________________________

These questions refer to the past <u>12 months</u>

---

31) Have you used drugs other than those required for medical reasons?	☐ Yes	☐ No
32) Do you abuse more than one drug at a time?	☐ Yes	☐ No
33) Are you always able to stop using drugs when you want to?	☐ Yes<br>☐ Never used drugs	☐ No
34) Have you had “blackouts” or “flashbacks” as a result of drug use?	☐ Yes	☐ No
35) Do you ever feel bad or guilty about your drug use?	☐ Yes<br>☐ Never used drugs	☐ No
36) Does your spouse (or family) ever complain about your involvement with drugs?	☐ Yes	☐ No
37) Have you neglected your family because of your use of drugs?	☐ Yes	☐ No
38) Have you engaged in illegal activities in order to get drugs?	☐ Yes	☐ No
39) Have you ever experienced withdrawal symptoms (felt sick) when you stopped taking drugs?	☐ Yes	☐ No
40) Have you had medical problems as a result of your drug use (e.g., memory loss, hepatitis, convulsions, bleeding, etc.)?	☐ Yes	☐ No
